# Enhancing iron and zinc bioavailability in maize (*Zea mays*) through phytate reduction: the impact of fermentation alone and in combination with soaking and germination

**DOI:** 10.3389/fnut.2024.1478155

**Published:** 2024-12-02

**Authors:** Samuel Nsabimana, Tariq Ismail, Claudia E. Lazarte

**Affiliations:** ^1^Division of Food and Pharma, Department of Process and Life Science Engineering, Faculty of Engineering, Lund University, Lund, Sweden; ^2^Food Science and Technology, University of Rwanda-College of Agriculture, Animal Sciences and Veterinary Medicine (UR-CAVM), Nyagatare, Rwanda; ^3^Department of Food Science and Technology, Faculty of Food Science and Nutrition, Bahauddin Zakariya University, Multan, Punjab, Pakistan

**Keywords:** maize, fermentation, soaking, germination, mineral bioavailability, phytates

## Abstract

**Introduction:**

Phytates are nutrient-binding compounds found mainly in cereals and legumes, which may significantly contribute to micronutrient malnutrition in regions where phytate-rich cereals, such as maize, are staple food.

**Objectives:**

This study investigated how maize fermentation, both alone and in combination with soaking and germination, can reduce phytate levels and enhance the estimated bioavailability of iron and zinc.

**Methods:**

We evaluated various fermentation methods, including spontaneous fermentation; fermentation with starter cultures, either *Lactiplantibacillus plantarum* 299v^®^ (Lp299) or yogurt containing viable *Lacticaseibacillus casei*; and fermentation with Lp299 of soaked and germinated maize. The outcome variables included changes in pH and lactic acid content during fermentation, and measurements of phytate levels (spectrophotometry), minerals (Atomic absorption) and protein (protein analyzer) in maize samples before and after treatments.

**Results:**

Fermentation with Lp299 of soaked and germinated maize grains yielded a phytate reduction of up to 85.6% decreasing from 9.58 ± 0.05 g·kg^−1^ in raw maize to 1.39 ± 0.09 g·kg^−1^ after processing. Fermentation of raw maize flour using Lp299 or yogurt resulted in a similar phytate reduction of 65.3% (3.35 ± 0.26 g·kg^−1^) and 68.7% (3.02 ± 0.01 g·kg^−1^) respectively. Spontaneous fermentation yielded a phytate reduction of 51.8% (4.65 ± 0.40 g·kg^−1^). This reduction in phytate content enhanced the estimated bioavailability of iron and zinc, particularly in the soaking-germination-fermentation combination, where the Phytate:Zinc molar ratio (Phy:Zn) dropped from 40.76 to 7.77, representing 81% reduction from the raw maize. The Phytate:Iron molar ratio (Phy:Fe) dropped from 41.42 to 6.24 indicating an 85% reduction. Additionally, fermentation led to a significant increase (*p* = 0.001) in protein content in maize flour after fermentation, ranging from 7.3 to 10.3% after the various fermentation treatments. There was not significant difference in the protein increase when compared the fermentation types.

**Conclusion:**

Lactic acid fermentation of soaked and germinated maize grains, emerged as the most promising process to enhance the bioavailability of essential minerals. This approach could help alleviate mineral deficiencies in populations dependent on maize-based diets. The findings underscore the potential of fermentation to be applied at the household level, which may bring up an alternative for programs and policies focused on reducing micronutrient deficiencies and improving food security in developing regions.

## 1 Introduction

Globally, iron and zinc deficiencies pose a public health concern. The severity of this problem is particularly high in developing countries, while deficiencies are also rising among vegetarian and vegan populations in developed countries. These nutritional inadequacies contribute significantly to adverse health outcomes, such as iron deficiency anemia, which affects nearly 1.2 billion people worldwide (1). Such deficiencies result in productivity losses due to impaired cognition, stunted physical growth, and increased susceptibility to infections, morbidity and mortality (2). In Rwanda, anemia prevalence among children under five has been reported to range from 46.2 to 52.79% (3, 4).

Eastern and Western Africa have the highest per capita consumption of maize (*Zea mays*) as a staple crop. In these regions, the per capita consumption of maize ranges from 157–267 g per person per day. At these consumption levels, maize is expected to meet the dietary requirements for essential macro and micronutrients for children and women in these regions (5). However, the delivery of essential elements such as iron and zinc from the maize-based food products is compromised due to high levels of phytates. This situation poses a significant risk of micronutrients malnutrition and associated health disorders for people consuming maize as a staple food. Nearly 80% of the minerals in maize kernels concentrate in the germ portion that also serves as storage site for phytates and drastically affects minerals bioavailability (6). Additionally, phytates interact with available proteins and amino acids, forming insoluble complexes, and thereby making them unavailable for absorption in the gastrointestinal tract (55).

Nearly 1–3% (w/w) phytate appears in all plant seeds. Based on the cultivars and crop cultivation conditions, the concentration of phytates in maize ranges from 2.77 to 16.70 mg·g^−1^ of seeds (7). The estimated impact of phytates on minerals bioavailability is measured by calculating phytates to mineral ratios. Phytates:Zinc (Phy:Zn) molar ratio below 15 and Phytates:Iron (Phy:Fe) molar ratio below 1 are considered adequate for optimal absorption of listed minerals (8). However, maize exceeds these molar ratio thresholds, contributing to higher risks of iron and zinc deficiency among regular consumers of maize and maize-based products (9, 10). Considering their nutrients binding properties, several strategies have been evolved to mitigate the levels of phytates in maize. These strategies include mineral biofortification, as well as breading and genetic engineering approaches to reduce phytate levels in cereals. These methods are being explored as effective alternatives to increase mineral content and bioavailability in cereals (11). Depending on the specific objectives, the latest techniques may involve lengthy development times, and their widespread adoption can take time. In addition, consumer perception and acceptance of improved crops play a crucial role in the success of biofortification programs. Food processing methods, such as enzymatic treatment, heat treatments like cooking and baking are also used to reduced phytates. However, each method has its limitations. For example, enzymatic methods can be expensive and there is limited availability of phytase for human consumption. We should also consider that phytates are heat stable, so heat treatments like cooking and baking have limited efficacy reducing phytates. Dehulling has also been used to decrease phytates, but removing the outer layers of cereals can also remove valuable nutrients, including fiber, vitamins and minerals that are concentrated in the outer layers of many foods (12).

Simpler and more effective processing techniques, such as soaking, germination and fermentation, have proven effective at degrading phytates and improving mineral bioavailability in various crops (13–15). Fermentation an ancient food preservation technique, can enhance various nutritional and sensory properties depending on the starter culture and fermentation conditions (16, 17). Fermentation has been effective in reducing phytate content in cereals and pseudo cereals. During fermentation, either microbial phytase production or the activation of endogenous phytase can effectively hydrolyse phytates, thereby enhancing bioavailability of divalent minerals (18). Similar enzyme activation and phytate hydrolysis have been observed with soaking and germination techniques (19). Phytates reduction in maize through malting, germination and fermentation has been reported to range from 28 to 96% (13, 20, 21).

Given the dietary significance of maize in African countries and the risk factors that can undermine its nutritional value, designing culturally acceptable interventions to improve the nutrient delivery of this staple crop is essential for enhancing nutrition security in vulnerable populations. Fermentation has great potential to enhance the nutritional value of foods. However, despite its benefits, the widespread use of fermentation for nutritional improvement remains limited. This is partly due to a lack of standardized methods, variability in microbial cultures, and limited understanding of how fermentation conditions affect both nutrient retention and degradation. More research is needed to optimize fermentation processes and integrate them into contemporary food production, ensuring that both nutritional improvements and sensory qualities are preserved. By doing so, we can unlock the untapped potential of fermentation to combat nutrient deficiencies and improve food security, particularly in regions where malnutrition is common.

The focus of this study was to evaluate convenient, affordable, culturally acceptable and feasible processes to reduce phytates. We compared soaking, germination and fermentation (spontaneous, with lactic bacteria and yogurt containing viable bacteria), both alone and in combination, to reduce phytate levels in maize. We also assessed changes in the estimated bioavailability of iron and zinc following each processing method. Additionally, since protein is a key nutrient, we measured changes in protein content resulting from the various treatments. This study represents an opportunity to apply maize fermentation at household level, providing a sustainable process to improve iron and zinc bioavailability in vulnerable populations. Moreover, the findings could inform national programs and policies aimed at reducing malnutrition and food insecurity in African countries.

## 2 Materials and methods

### 2.1 Materials and sample preparation

We ordered the maize grains for this study from the Rwanda Agriculture Board (RAB), with the biological identification code ZM607 - MUTUTU-18A basic. We selected ZM607 - MUTUTU-18A for its high productivity in the lowlands commonly used for maize production. We purchased *Lactiplantibacillus plantarum 299v^®^* (Probi, Lund, Sweden) from a pharmacy in Lund, Sweden, and a commercial drinkable yogurt containing 20 billion *Lacticaseibacillus casei* starter culture (Actimel, Danone, France) from a supermarket (ICA, Lund, Sweden). We conducted all the experiments in Sweden.

**Figure 1:**
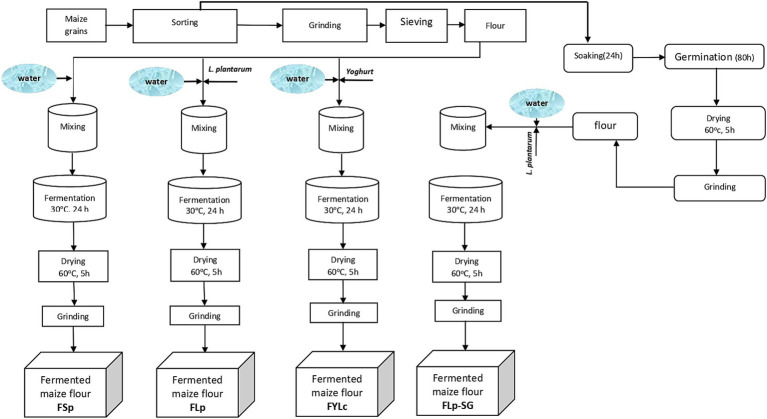
Flow chart of processing steps to obtain fermented maize flour under different conditions. FSp, Spontaneous fermentation of maize flour; FLp, Fermentation of maize flour with *L. plantarum* 299v^®^; FYLc, Fermentation of maize flour with yoghurt containing viable *L. casei*; FLp-SG, Fermentation of maize flour from soaked and germinated maize kernels with *L. plantarum* 299v as starter culture.

We manually sorted the maize grains to remove damaged grains and other extraneous materials. We then divided the grains into two batches for further processing. For the first batch, we milled the grains into fine flour using a laboratory hammer mill fitted with a 0.5 mm sieve. The milled samples were packed in airtight plastic bags and stored at 4°C for further processing and analysis. This first batch was used for three different fermentation processes: spontaneous fermentation, fermentation with *L. plantarum 299v^®^* (Lp299), and fermentation with yogurt containing viable *L. casei.* For the second batch, we soaked and germinated the grains. After germination, grains were ground and sieved through a 0.5 mm sieve to obtain flour. This flour from soaked and germinated grains was then fermented with Lp299. We conducted the experiments in two independent runs. A detailed description of the processes is provided below, and Figure 1 presents a schematic representation of all steps and conditions used during the processes.

### 2.2 Soaking and germination

We soaked the maize grains in deionized water at a 1:3 (w/v) ratio at room temperature (18°C) for 24 h in jars, using static soaking without water changes, as described by Mihafu et al. (22). After soaking, we drained off the soaking water using an absorbent cloth and moved the soaked grains to the next step of germination. We took a sample of the soaked grains, wet ground it, and dried it at 105°C until it reached a constant weight, then stored it for further analysis.

We followed the method described by Mihafu et al. (22) for germination with some modifications. Instead of baskets, we used well-prepared plastic containers for germinating the soaked maize grains. We placed a moistened cotton cloth (0.25 m × 0.25 m) inside the container folding it to cover the base circumference. The soaked maize grains were spread on the cloth and were covered with another moistened cloth of the same length. To protect the grains from light and maintain warmth for sprouting, the container was covered with a black cloth. The maize grains were left to germinate for 80 h at room temperature (18°C) in Sweden, rather than 72 h, as the lower room temperature required a longer germination time compared to the original research conducted in Tanzania, where the room temperature was around 25°C. After germination, we spread the maize grains on baking paper and dried them in an oven (Termaks, Lund, Sweden) at 60°C for 5 h. We then ground the dried germinated maize grains using a laboratory hammer mill (Laboratory Mill 120, Lund, Sweden) and sieved the flour through a 0.5 mm sieve to obtain a fine consistency. The milled flour samples were packed in airtight plastic bags and stored at 4°C for further analysis and use in the fermentation process.

### 2.3 Fermentation, spontaneous and with starter culture

We followed the fermentation process described in our previous research (16, 18, 23) with some modifications. The modification in the current study included reducing the fermentation time to 24 h instead 48 h and using yogurt as starter culture. Briefly, a suspension of 500 g of maize flour was prepared in de-ionized water in plastic containers at the ratio of 1:2 (w/v). For each type of fermentation, we divided the slurry into two portions and placed them in separate hermetically sealed containers to ferment in replicates. Prior to incubation, we cultured the samples using either spontaneous or inoculated fermentation methods vis. Spontaneous fermentation of maize flour from raw grains (FSp); Lp299 led fermentation of maize flour developed from raw grains (FLp); lactic acid fermentation of maize flour with yogurt containing viable *L. casei* (FYLc); and fermentation with Lp299 of maize flour from soaked and germinated maize kernels (FLp-SG).

For FLp, we dissolved Lp299 (7.35 Log10 CFU·g^−1^) at a proportion of 1% in distilled water before mixing it with maize flour and water. FYLc involved inoculating 5% yogurt (containing 20 billion *L. casei* cultures) by dissolving the inoculum in distilled water and mixing it with 500 g of maize flour at a 1:2 (w/v) ratio. For FLp-SG, we dissolved Lp299 in distilled water and mixed it with 500 g of maize flour obtained from soaked and germinated maize grains, maintaining the same 1:2 (w/v) ratio.

We incubated the fermentation containers at 30°C for 24 h, taking samples of the slurry every 4 h from 0 to 12 h, with a final sample taken at 24 h. We analyzed each sample for pH, titratable acidity (as lactic acid percentage), dry matter, and phytate content. At the end of the fermentation process, we decanted the fermentation water and transferred the samples to aluminum foil for drying in the oven (Termarks, Lund, Sweden) at 60°C for 5 h. The dried samples were then ground in a hammer mill and stored at 4°C for further analysis of phytate, protein, and mineral content.

### 2.4 Chemical analyses

#### 2.4.1 Determination of moisture content

We determined the moisture content in maize flour in duplicates by following standardized procedures (18). Briefly, we transferred 5 g (±0.0001 g) of maize flour into dried and weighed dishes, then placed the samples in an oven at 105°C until they reached a constant weight. We computed the moisture content of the samples using a differential method.

#### 2.4.2 pH and acidity determination

We determined the pH and acidity of fermented samples in duplicate, withdrawing samples every 4 h until the pH dropped below 4.6, following the method described in Castro-Alba et al. (18). Briefly, we suspended 10 g of each sample in 90 mL of de-ionized water and stirred the mixture for 10 min. We then filtered the suspension and measured the pH of the liquid by dipping the pH electrode (Metrohm 744 pH meter, Switzerland) into the homogenized mixture.

In addition to pH, we also determined the total acidity of the withdrawn samples in duplicate. We suspended 10 g of the fermented slurry sample in 90 mL of de-ionized water and stirred it for 10 min. Next, we titrated 75 mL of the homogenized sample against 0.1 N NaOH using a 1% phenolphthalein indicator. We recorded the volume of 0.1 N NaOH used for titration. We expressed the total acidity as g·kg^−1^ DM of lactic acid, where 1.0 mL of 0.1 N NaOH was equivalent to 9.0 × 10^−3^ g of lactic acid.

#### 2.4.3 Determination of phytate content

We determined phytate following the method described by Makkar et al. (24) and the modifications presented by Ayub et al. (16). Briefly, we mixed 1.5 g of maize flour with 50 mL of extracting solution (3.5% HCl) in a 200 mL volumetric flask and stirred the mixture vigorously with a magnetic stirrer at 500 rpm for 60 min at room temperature. We then centrifuged the mixture at 3,000 g for 10 min at 20°C (Beckman Coulter Allegra x-15r, UK), and diluted 5 mL of the supernatant to 25 mL with distilled water.

To purify the phytate, we used an anion exchange column (200–400 mesh). We prepared duplicates of 0.7 cm × 15 cm columns, plugging them with a small quantity of cotton wool. We vertically fixed the columns and filled them with 0.5 g of AGI-X8 chloride anion-exchange resin (DOWEX^®^1×8 Chloride form, 100–200 mesh, Sigma) to separate inorganic phosphorus and other interfering compounds from inositol phosphates. We allowed 10 mL of diluted sample extracts to pass through the column. After that, we transferred 15 mL of 0.1 M NaCl into the column to elute the inorganic phosphorus and other interfering compounds, followed by 15 mL of 0.7 M NaCl to elute the phytate. We discarded the resin in the column after use.

Next, we added 1 mL of Wade reagent, made from 30 mg of FeCl₃·6H_2_O and 357 mg of sulfosalicylic dihydrate, to 3 mL of the eluted sample for phytate analysis and vortexed the mixture for 5 s. We centrifuged the homogeneous mixture at 3,000 g for 10 min at 20°C (Beckman Coulter Allegra x-15r, UK). We recorded the absorbance values of the samples at 500 nm (Varian 50 Bio UV–Visible Spectrophotometer, Hamburg, Germany) against a reagent blank. We prepared a series of standard solutions containing 5, 10, 15, 30, and 50 μg·mL^−1^ of phytic acid in distilled water for quantification. We presented the results on a dry matter basis.

#### 2.4.4 Determination of mineral content

We determined the mineral content in both unfermented and fermented maize flour following the procedure described by Lazarte et al. (10). We washed all glassware and materials used during the analysis with a 3% nitric acid solution to avoid contamination, then double-rinsed them with de-ionized water. Next, we placed approximately 0.5 g of each sample in Teflon vessels, mixing it with 2 mL of H_2_O_2_ (30% v/v) and 3 mL of HNO_3_ (65% v/v). We tightly closed the vessels and performed acid digestion for one hour in a microwave reaction system (MRS5, Microwave Accelerated Reaction System, CEM, Matthews, NC, USA). After digestion, we diluted each sample to 25 mL with de-ionized water. We measured iron and zinc using Flame Atomic Absorption Spectrometry with an air-acetylene flame (Agilent Technologies 200 Series AA, Agilent, Santa Clara, CA, United States) at wavelengths of 213.9 and 248.3 nm, respectively. We prepared a 5-point calibration curve in the range of 100–2,000 mg·L^−1^ using certified Atomic Absorption Standard solutions for iron and zinc (Merck KGaA, Darmstadt, Germany).

#### 2.4.5 Estimation of mineral bioavailability

We estimated iron and zinc bioavailability in both unfermented and treated samples using the molar ratios of phytate to mineral. We used a molar weight of 660 g·mol^−1^ for phytate. We then compared the Phytate:iron (Phy:Fe) and Phytate:zinc (Phy:Zn) of the samples to the recommended values for adequate iron and zinc bioavailability, where Phy:Fe should be less than 1 and Phy:Zn less than 15 (25).

#### 2.4.6 Determination of protein contents in raw and processed maize flours

We determined the protein content of the unfermented and fermented samples using the Dumas (dynamic flash combustion) method (26) with a protein analyzer (Thermo Scientific™ FLASH™, EA 1112 series, United States). This analyzer detects the amount of nitrogen present in the sample after complete combustion. We then multiplied the nitrogen amount by a nitrogen-to-protein conversion factor of 6.25. We present the results of crude protein content in each sample on a dry matter basis.

### 2.5 Statistical analysis

We conducted all fermentation trials in duplicate, withdrawing two samples from each batch at each sampling time to analyze various parameters. We used the Kolmogorov–Smirnov test to evaluate the normality of the data, confirming that the datasets followed a normal distribution. As a result, we presented the data as mean values ± standard deviation and employed parametric tests for further statistical analysis. We performed a one-way ANOVA to assess the effects of fermentation type on pH, lactic acid content, iron, zinc, phytate, and phytate molar ratios. Following ANOVA, we used Tukey’s *post-hoc* test for multiple comparisons to determine significant differences between the means of the four fermentation types, with the significance level set at *p* < 0.05. A two-way ANOVA was computed to investigate the effect of the interaction between fermentation type and fermentation time (for samples withdrawn at 0, 4, 8, 12, and 24 h fermentation) on the response variables phytate, pH, lactic acid and protein content. This was followed by multiple pairwise comparisons to identify significant effects at each sampling time.

Additionally, we conducted another round of one-way ANOVA, followed by Tukey’s test, to assess the effects of soaking and germination on phytate, mineral content, and molar ratios. This analysis aimed to elucidate the changes due to the pre-treatments of soaking and germination. We used SPSS Statistics 24 (SPSS Inc., IBM Corporation, Armonk, United States) for all calculations.

## 3 Results

### 3.1 Moisture contents in raw and processed maize flours

We observed a moisture content of 10.6% in raw maize flour. After drying at 60°C for 5 h, the moisture content in spontaneously fermented flour (FSp) was 6.94%, in flour fermented with Lp299 (FLp) it was 6.13%, in flour fermented with yogurt as starter culture (FYLc) it was 6.49%, and in fermented flour from soaked and germinated maize grains (FLp-SG) it was 4.91%.

### 3.2 Effect of fermentation on pH and lactic acid

**Figure 2:**
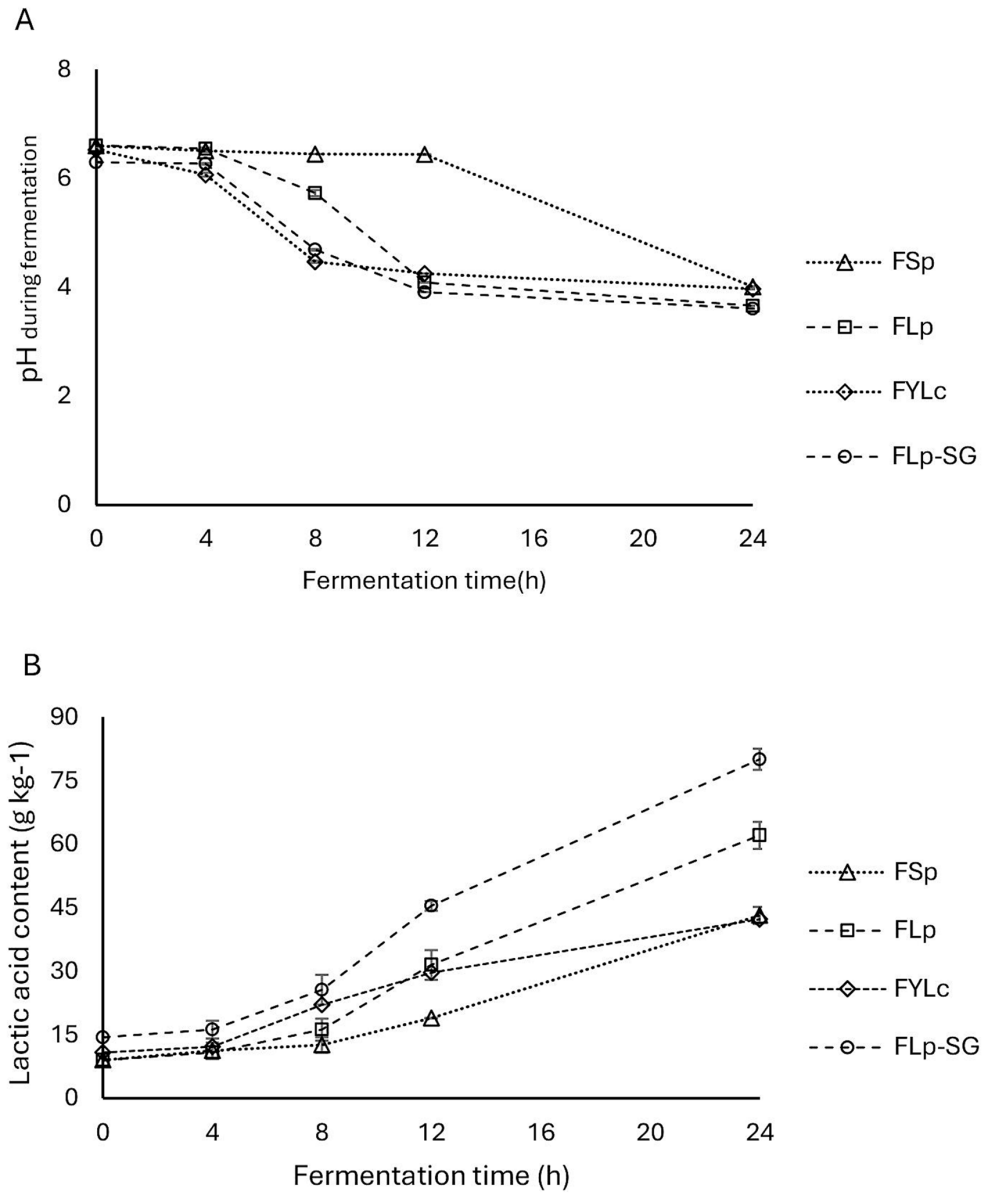
Changes in pH and lactic acid during fermentation. FSp, Spontaneous fermentation of maize flour; FLp, Fermentation of maize flour with *L. plantarum* 299v^®^; FYLc, Fermentation of maize flour with yoghurt containing viable *L. casei*; FLp-SG, Fermentation of maize flour from soaked and germinated maize kernels with *L. plantarum* 299v as starter culture.

We found a decrease in pH and a relative increase in lactic acid content across all four fermentation types: FSp, FLp, FYLc, and FLp-SG, as shown in Figures 2A,B. The initial pH of the first two fermentations, FSp and FLp, was nearly identical at 6.60 and 6.61, respectively. Lactic acid levels in both samples were 9.0 g·kg^−1^. A slight difference was observed in the FYLc sample at time 0, where the pH was 6.53 and lactic acid was 10.8 g·kg^−1^. The fermented sample made from soaked and germinated maize kernels (FLp-SG) showed a lower initial pH of 6.31 and a higher titratable acidity expressed as 4.4 g·kg^−1^ of lactic acid. This indicates that soaking and germination lowered the pH and increased the lactic acid content even before fermentation began.

During the first 4 h of fermentation, the changes in pH across the four fermentation types were not significant (*p* > 0.05). From 4 to 12 h, we observed a more pronounced pH decrease in the maize slurry fermented with yoghurt (FYLc) and in the maize slurry from soaked and germinated kernels fermented with Lp299. Meanwhile the spontaneous fermentation group (FSp) showed no obvious pH change during this period, with a pH of 6.44 after 12 h. Between the 12 to 24 h of fermentation, all groups containing starter culture showed pH stabilization, with final pH values 3.66 for FLp, 3.97 for FYLc and 3.61 for FLp-SG, respectively. In contrast, the spontaneous fermentation showed a significant pH drop in the last 12 h, with the pH decreasing from 6.44 to 4.01 (Figure 2A).

During the fermentation period, lactic acid content steadily increased, with FLp-SG showing the most significant rise. By the end of fermentation, FLp-SG reached 80.1 g·kg^−1^ of lactic acid. Comparing the lactic acid content obtained from maize flour fermented with Lp299 and maize flour fermented with yogurt, FLp reached 62.1 g·kg^−1^, while FYLc measured 42.3 g·kg^−1^ lactic acid. Between 0 and 8 h of fermentation, we observed a significant reduction in pH and a slower increase in lactic acid in the samples fermented with yogurt, compared to those fermented with Lp299. In samples that underwent spontaneous fermentation, lactic acid content increased from 9.0 to 18.9 g·kg^−1^ in the first 12 h. Whereas the rate of increase surged by approximately 129% over the next 12 h, resulting in lactic acid levels rising from 18.9 to 43.2 g·kg^−1^ (Figure 2B).

The results from the two-way ANOVA test showed that both pH and lactic acid content were significantly affected (*p* = 0.000) by fermentation time (4, 8, 12, and 24 h) and fermentation type (FSp, FLp, FYLc, and FLp-SG). The effect of fermentation time on pH and lactic acid was significant at *p* = 0.000, as was the effect of fermentation type. Moreover, the interaction between these two factors-time and type of fermentation-had a statistically significant effect (*p* = 0.000) on the response variables. Figure 2A (pH) and Figure 2B (lactic acid) show the interaction plots of fermentation time and type, illustrating the impact of both factors on pH and lactic acid content.

A post-hoc analysis of multiple comparisons revealed similar trends for pH and lactic acid content. At time 0, there was no significant difference in pH and lactic acid content between the maize samples used in FSp, FLp, and FYLc (*p* = 0.999). However, these samples significantly differed from the FLp-SG sample (*p* = 0.005), the difference can be attributed to the soaking and germination steps prior to fermentation. At 4 h, lactic acid content in FSp was not significantly different from that in FLp (*p* = 0.793), FYLc (*p* = 0.600) but differed significantly from FLp-SG (*p* = 0.005). At 8 h fermentation, lactic acid content in FSp showed a slight but significant difference from that in FLp (*p* = 0.046), with a more pronounced significance level when comparing FSp to FYLc (*p* = 0.002) and FLp-SG (*p* = 0.000). At 12 and 24 h fermentation, lactic acid content was significantly different across all fermentation types (*p* = 0.000), except between FLp and FYLc where the *p*-value was 0.299 at 12 h and 0.600 at 24 h fermentation.

### 3.3 Effect of soaking, germination, and fermentation on phytate content

Our results indicate that 24 h soaking, and 80 h germination reduced phytate content in maize flour from 9.65 g·kg^−1^ to 8.44 g·kg^−1^ and 6.57 g·kg^−1^, respectively. The rate of phytate reduction after soaking was 12.6%, and after germination, it was 31.9%, indicating that germination is a promising and simple technique for reducing phytate levels to some extend (Table 1).

**Table 1:** Phytate, minerals and estimated mineral bioavailability before and after processing, results are presented as mean ± SD in dry weight.

Parameters	Unfermented maize	FSp	FLp	FYLc	Soaked maize	Germinated maize	FLp-SG
Moisture (%)	10.6 ± 0.11	6.94 ± 0.93	6.13 ± 0.15	6.49 ± 0.88	8.81 ± 0.15	10.15 ± 0.91	4.91 ± 0.21
Phytate (g·kg^−1^)	9.58 ± 0.05^fD^	4.65 ± 0.40^cC^	3.35 ± 0.26^bB^	3.02 ± 0.01^bB^	8.44 ± 0.06^e^	6.35 ± 0.03^d^	1.39 ± 0.09^aA^
Zinc (mg·kg^−1^)	21.10 ± 0.89^bB^	19.73 ± 0.50^abAB^	18.33 ± 0.49^abA^	19.57 ± 0.49^abAB^	19.63 ± 2.01^ab^	20.53 ± 0.45^ab^	17.73 ± 0.68^aA^
Iron (mg·kg^−1^)	19.57 ± 0.60^aA^	19.67 ± 1.26^aA^	18.07 ± 1.47^aA^	17.70 ± 1.47^aA^	18.43 ± 0.81^a^	18.17 ± 1.14^a^	18.87 ± 1.19^aA^
Phy:Zn	40.76 ± 2.36^eE^	23.42 ± 0.49^cD^	18.14 ± 0.82^bcC^	15.30 ± 0.32^bB^	40.57 ± 1.29^e^	30.65 ± 0.55^d^	7.77 ± 0.24^aA^
Phy:Fe	41.42 ± 1.27^eD^	19.97 ± 1.16^cC^	15.68 ± 0.32^bB^	14.49 ± 1.27^bB^	38.76 ± 1.75^e^	29.63 ± 1.90^d^	6.24 ± 0.39^aA^
% of phytate reduction		51.8 ± 4.10	65.3 ± 0.26	68.7 ± 3.2	12.6 ± 0.6	31.9 ± 1.9	85.6 ± 0.91

For each parameter (each row), superscripts lowercase letters (a, b, etc.) indicate differences between untreated maize, and after the treatments; soaking, germination and all fermentations. Superscripts uppercase letters (A, B, etc.) indicate differences due to fermentation types against the untreated maize. Results are presented as mean ± SD and significant differences were computed at *p* < 0.05. FSp, Spontaneous fermentation of maize flour; FLp, Fermentation of maize flour with *L. plantarum* 299v^®^; FYLc, Fermentation of maize flour with yoghurt containing viable *L. casei*; FLp-SG, Fermentation of maize flour from soaked and germinated maize kernels with *L. plantarum* 299v as starter culture.

**Figure 3:**
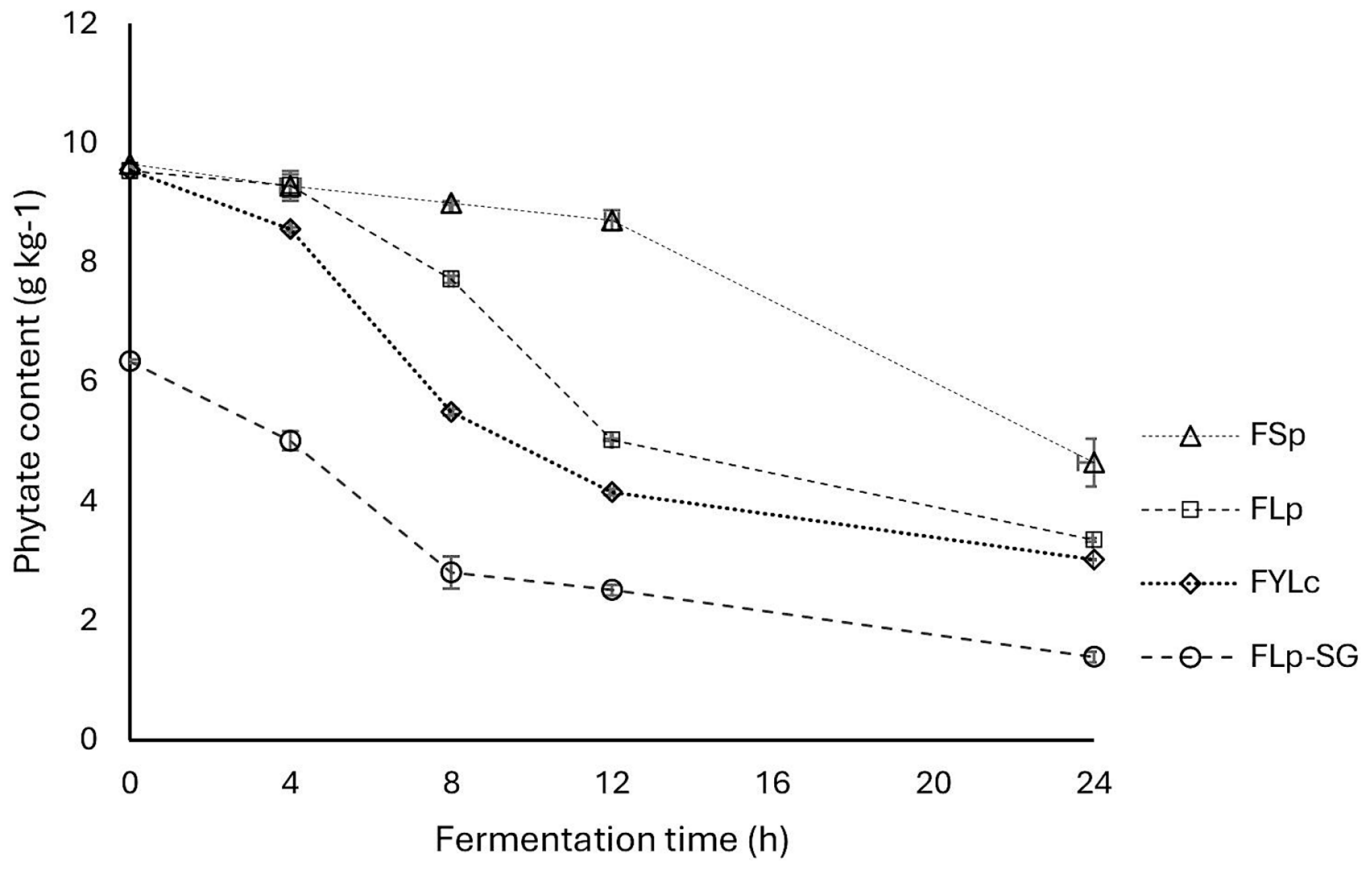
Changes in phytate content through various fermentation processes of maize flour. FSp, Spontaneous fermentation of maize flour; FLp, Fermentation of maize flour with *L. plantarum* 299v^®^; FYLc, Fermentation of maize flour with yoghurt containing viable *L. casei*; FLp-SG, Fermentation of maize flour from soaked and germinated maize kernels with *L. plantarum* 299v as starter culture.

This study found a significant (*p* = 0.000) reduction in phytate content after all types of fermentation, with notable differences between fermentation types due to variations in starter cultures and pretreatment methods like soaking and germination. The changes in phytate content are shown in Figure 3. After 24 h of fermentation, phytate reductions were as follows: 51.8 ± 4.10% for FSp, 65.3 ± 0.26% for FLp, 68.7 ± 0.13% for FYLc, and 85.6 ± 0.91% for FLp-SG. During FSp, the phytate reduction was slow in the first 12 h, followed by a rapid drop in final 24 h, as shown in Figure 3. The process that led to the highest phytate reduction was FLp-SG, with an 85.60% reduction at 24 h, achieved through fermentation of maize flour made from soaked and germinated kernels. These pre-treatments—soaking and germination—significantly enhanced phytate reduction. This phytate reduction was followed by that obtained in FYLc 24 h, where fermentation with yoghurt culture reduced phytate by 68.7%, followed by FLp 24 h with LP299 starter (65.3%), and FSp 24 h, which showed the lowest reduction at 51.8%.

The varying effectiveness of the fermentation processes in reducing phytate levels can be attributed to pretreatments like soaking (24 h) and germination (80 h) and the effectiveness of the starter cultures. No significant difference was observed between FLp and FYLc in phytate reduction, suggesting that Lp299 and *L. casei* are similarly effective in reducing phytate.

The results from the two-way ANOVA test showed that the phytate content in fermented maize samples was significantly affected (*p* = 0.000) by both time (4, 8, 12, and 24 h) and fermentation type (FSp, FLp, FYLc, and FLp-SG). Both factors had a statistically significant effect on phytate content, with *p* = 0.000 for fermentation time and *p* = 0.000 for fermentation type. Additionally, the interaction between the two factors also had a statistically significant effect (*p* = 0.000) on the response variable. Figure 3 shows the interaction plot of the two variables to better visualize how both factors affect phytate content.

Moreover, a post-hoc analysis of multiple comparisons (pairwise comparisons) revealed that at time 0, there was no significant difference in phytate content between the maize samples used in FSp, FLp, and FYLc (*p* = 0.392). However, these samples were significantly different (*p* = 0.000) from the FLp-SG sample at time 0 h, due to the soaking and germination of the FLp-SG sample prior to fermentation. At subsequent time points (4, 8, 12, and 24 h), there was a significant difference (*p* = 0.000) in phytate content based on the fermentation type.

### 3.4 Effect of soaking, germination, and fermentation on mineral content

Table 1 presents the mineral content of raw maize flour and fermented maize flour obtained after all processes (i.e., soaking, germination and fermentation). We observed a slight non-significant decrease in zinc content during FSp, FLp, and FYLc by 6, 13, and 7%, respectively. However, we found a significant lower zinc content after FLp-SG, where the concentration dropped from 21.10 to 17.73 mg·kg^−1^, representing a16% decrease. This reduction can be attributed to the pre-treatment of soaking, where zinc may have leached into the discarded soaking water. There were no significant differences in the iron content before and after treatments.

### 3.5 Mineral bioavailability estimated by the phytate: mineral molar ratios

The recommended phytate to mineral molar ratios for adequate bioavailability of zinc and iron are Phytate:Zinc (Phy: Zn) <15 and Phytate:Iron (Phy: Fe) <1. In this study, the calculated molar ratios for and Phy:Fe and Phy:Zn, before and after treatments, are presented in Table 1. Before any treatment, the molar ratios in maize were significantly above the recommended values, with a Phy:Zn of 40.76 and a Phy:Fe of 41.42. All fermented maize flours showed improved phytate:mineral molar ratios, although most remain above the threshold values. The exception was the Phy:Zn of 7.77, found in the fermented maize flour developed from pre-soaked and germinated grains (FLp-SG). Compared to the Phy:Zn of raw maize, there was a substantial reduction from 40.76 to 7.77 (an 81% decrease), which fell well below the threshold level of 15. This result implies that zinc bioavailability in maize was no longer inhibited by phytates after the treatment FLp-SG. Phy:Zn ratio for spontaneously fermented flour also improved from 40.76 to 23.42, although it remained above the threshold of 15. Other fermentation treatments such as FLp and FYLc yielded comparable Phy:Zn ratio of 18.14 and 15.30, respectively, representing reductions of 55 and 62%, respectively, from the raw maize.

Phy:Zn ratios for only soaked or soaked-germinated maize were 40.57 and 30.65 respectively, indicating that these techniques alone were insufficient to significantly improve zinc bioavailability. Among all fermentation types, the FLp-SG process proved most effective at bringing Phy:Zn molar ratios below the recommended level 15, indicating that zinc bioavailability in this case was not longer impaired by phytate.

Phy:Fe molar ratio for untreated maize was 41,42. Soaked alone had minimal impact, reducing the ratio to 38.76, while germination brought it down to 29.63. In contrast, the FLp-SG treatment led to a substantial 85% reduction, lowering the Phy:Fe ratio to 6.24. This improvement was followed by FYLc (Phy:Fe ratio of 14.49), FLp (15.68) and FSp (19.97). Although none of the fermentation methods achieved the ideal phy:Fe molar ratio of below 1. Nevertheless, all the reductions were significant, and indicate potential for further enhancement of both fermentation methods and pre-treatments to improve iron bioavailability.

### 3.6 Effect of fermentation on protein content

**Figure 4:**
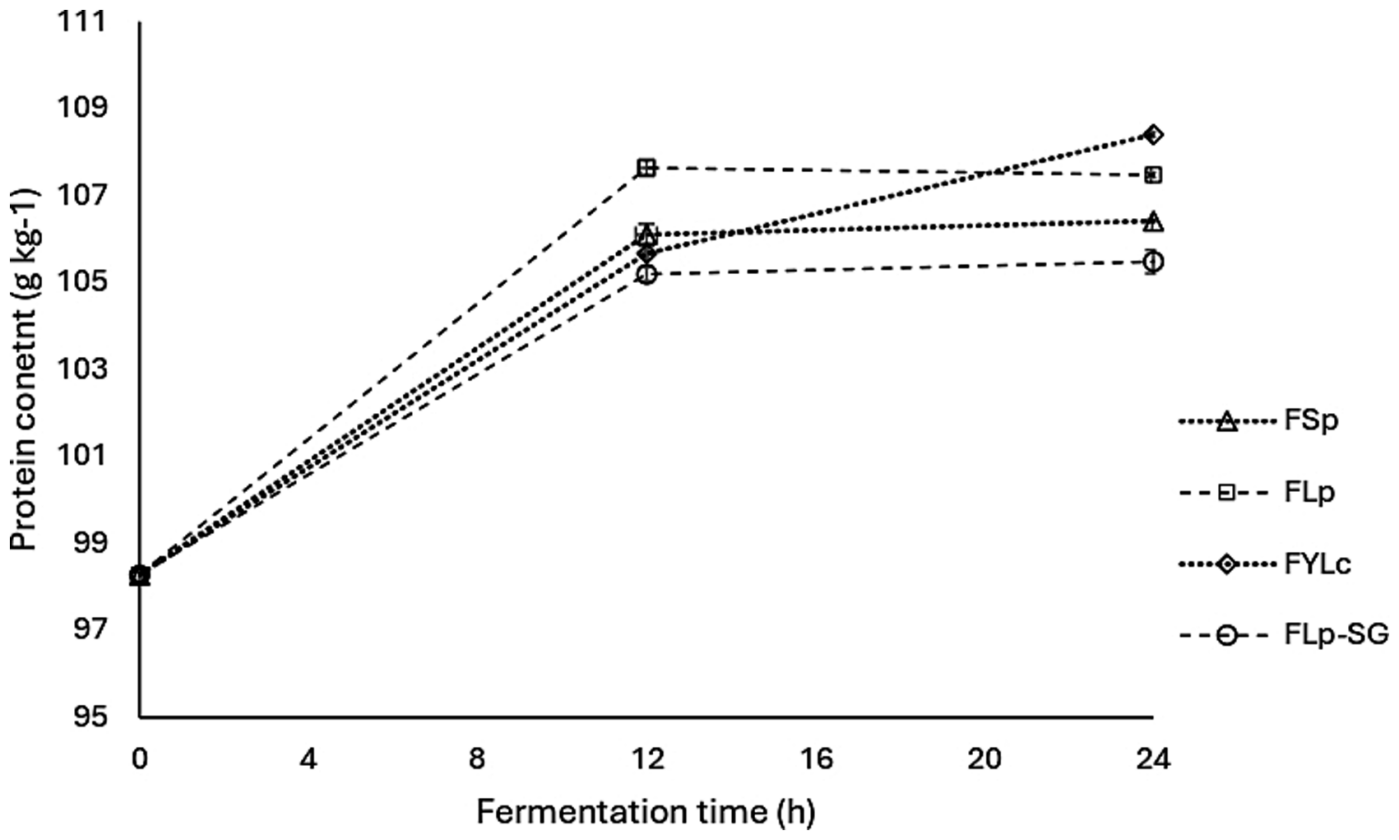
Changes in protein content during maize flour fermentation. FSp, Spontaneous fermentation of maize flour; FLp, Fermentation of maize flour with *L. plantarum* 299v^®^; FYLc, Fermentation of maize flour with yoghurt containing viable *L. casei*; FLp-SG, Fermentation of maize flour from soaked and germinated maize kernels with *L. plantarum* 299v as starter culture.

Figure 4 shows the crude protein content of maize flour determined at 0, 12, and 24 h of fermentation for all the four fermentation types. During the study, protein content increased at varying rates as fermentation time progressed for all the four fermentation types. Maize flour that underwent spontaneous fermentation for 24 h (FSp) showed a slight protein content increase of 8.2% from the baseline. Compared to the control or raw maize flour, protein content significantly increased (*p* < 0.05) by 9.3% in FLp, 10.3% in FYLc and 7.3% ^in^ FLp-SG.

The results from the two-way ANOVA test showed that protein content was significantly affected by fermentation time (*p* = 0.000) at intervals of 0, 12 and 24 h, but was not significantly affected by the fermentation type (*p* = 0.459) among FSp, FLp, FYLc, and FLp-SG. The interaction between the two factors – fermentation time and type-was also not statistically significant (*p* = 0.756) for protein content. Figure 4 presents the interaction plot of the two variables, providing visual representation of how both factors impacted protein content. Furthermore, the post-hoc analysis of multiple comparisons indicated that at time 0, 12 and 24 h, protein content was not significantly different across samples FSp, FLp, FYLc and FLp-SG with *p*-values of 0.000 at 0 h, ranging from 0.163 to 0.801 at 12 h, and from 0.097 to 0.569 at 24 h. However, protein content showed significant differences due to fermentation time; at 0 h, protein content was significantly lower (*p* = 0.000) than that recorded at 12 h, after which it did not significantly change (*p* = 0.595) until 24 h fermentation. This trend was consistent across all fermentation types.

## 4 Discussion

### 4.1 Effect of fermentation on pH and lactic acid

The reduction of pH during fermentation can be attributed to the lactic acid bacteria added as Lp299 or *L. casei* in yoghurt, these lactic bacterium converts available carbohydrates to organic acids that are responsible for the pH reduction and lactic acid increase. It is reported that in spontaneous fermentation, endogenous microbial activity reduces the substrate’s pH by using carbohydrates and producing organic acids such as lactic, citric, and acetic acids (27). In our study, relatively lower rate of reduction in pH and corresponding slight increase in lactic acid contents during first 12 h of spontaneous fermentation resulted from the time needed for endogenous microbes to activate and adapt to the fermentation conditions. The higher lactic acid content in FYLc during the first half of the fermentation period likely resulted from the lactic acid already present in the yogurt. After 8 h, the faster increase in lactic acid production in samples fermented with Lp299 strains can be due to the depletion of available carbohydrates, caused by the higher metabolic activity of the microflora during the initial phase of fermentation.

The results on the effect of fermentation on pH and the lactic acid contents of the substrate align with previous research. For instance, earlier studies reported that maize flour fermentation anticipated considerable pH reduction from 6.67 to 3.85 and total acidity increased from 37.97 to 71.59 g·kg^−1^ (28). Our study recorded a slightly higher pH of 4.01. Unlike the 129-h fermentation reported by Beugre et al. (28), we achieved this pH reduction in just 24 h, which highlights the potential for reducing fermentation time in cereals and other complex plant foods. Similarly, Ejigui et al. (29) found a 37% decrease in pH after 96 h of maize fermentation. While we observed a 39% reduction in 24 h. These differences can be attributed to the crop cultivar, grain size and fermentation conditions such as time, temperature and starter cultures. Improved microbiological activity to support lactic acid fermentation, decrease pH and increase corresponding lactic acid contents has also been endorsed by Wedad et al. (30) during 16 h sorghum fermentation.

Our study recorded a rapid 79% increase in lactic acid content from the baseline for 24 h fermentation, which closely aligns with the findings of Beugre G. et al. (28), where titratable acidity increased by 87% over 48 h fermentation. We observed a significant pH decrease (*p* < 0.001) and a corresponding significant increase in lactic acid content (*p* < 0.001) (Figure 2B). Among the fermentation processes, maize flour fermented with Lp199 after soaking and germination showed the greatest pH reduction (from 6.61 to 3.61) and lactic acid content increase (from 9.0 to 80.1 g·kg^−1^) (Figures 2A,B). This improved performance can be attributed to the early activation of endogenous microflora during soaking and germination of the maize kernels. Soaking and germination resulted in a slight drop in pH and an increase in lactic acid content. Differences between spontaneous fermentation and those using Lp299 or yogurt starter cultures can be linked to the predefined inoculation of the starter cultures, which supports the rapid activation of fermentation (31, 32).

The extended shelf life of fermented foods is largely linked to higher lactic acid contents and significant drop in pH, which inhibit spoilage bacteria and other pathogens. This also enhance the quality and consumer acceptability of fermented products. Achieving high lactic acid concentrations or a rapid pH decline to around 4.0 is highly desirable for preventing microbial spoilage and extending the shelf stability properties of maize flour (33). This quality is especially important when preparing maize-based weaning formulas for infants and young children, as it reduces the risk of spoilage or microbial contamination (31).

### 4.2 Effect of soaking and germination on phytate content

The results indicate that the phytate content in raw maize was consistent with previous studies, which reported levels ranging 7–14 mg·g^−1^ (27, 34). Phytate reduction in cereal products during soaking is attributed to the solubility of phytate in water. Since phytate is water soluble, soaking facilitates its reduction. In our study, phytate reduction during soaking was modest (12.6%), similar to findings by Kruger et al. (34), who reported a 14% reduction in whole white maize grains after soaking for 12 h at 25°C. Other authors have found that soaking removed up to 21% of phytate from maize grains (35). Previous studies have shown that the impact of soaking on phytate content depends not only on soaking conditions – such as pH, time, volume of soaking water and temperature – but also to the grain variety and state of the soaked grain. Hotz and Gibson (27) observed a 43% reduction of phytate after 12 h soaking of unrefined white maize flour at 25°C and a higher reduction (49%) when excess water was decanted. In their study, the ratio of maize to water was 1:4, and the soaking temperature was higher than in our present research. Duhan et al. (36) observed a 29% reduction in phytate from whole millet after 24 h of soaking, compared to 39 and 52% for once-dehulled millet and millet flour, respectively. These results indicate a greater potential for reducing phytate content by processing grains into flours rather than soaking intact grains. This demonstrates that increasing the surface area of seeds or grains enhances exposure to the soaking medium, improving the removal of soluble phytates.

It is important to note that soaking is a common pre-treatment method used to enhance the nutritional quality of pulses, legumes, and grains. While soaking can reduce anti-nutritional factors like phytates, it can also lead to the loss of some water-soluble nutrients. Nutrients such as B-complex vitamins (thiamine, riboflavin, niacin, and folate), vitamin C, minerals (potassium and magnesium), and soluble proteins can leach into the soaking water (37). Therefore, it is important to assess nutrient loss when evaluating soaking as a processing method.

The results of 80 h germination of maize grains showed a 31.9% reduction in phytate content. This result agrees with findings by Mihafu et al. (22) in Tanzania, where phytate reduction ranged from 8.3 to 34.1% after 72 h of maize germination. Additionally, Coulibaly et al. (38) reported a 23.9% phytate reduction in millet after 72 h of germination, with 96 h of malting leading to a 45.3% reduction. The reduction in phytate during germination is primarily attributed to phytate hydrolysis triggered by the activation of phytase enzymes, which increase during germination (39). These enzymes break down phytate, releasing phosphorus, myo-inositol, and minerals that serve as nutrients for the growing seedling. Other factors contributing to phytate hydrolysis include the species and varieties of the grains, temperature, moisture content, pH, duration of germination, phytate solubility, and the presence of inhibitors like tannins in sorghum (40).

### 4.3 Effect of fermentation on phytate content

Our statistical comparison using one-way ANOVA for different fermentation techniques revealed significant differences (*p* = 0.000) in their effectiveness at reducing phytate content in maize after 24 h fermentation (Figure 3). Furthermore, the two-way ANOVA test confirmed that, in addition to fermentation type, fermentation time also plays a significant role in reducing phytate content. While reduction of phytate was slower in the first 4 h, it was intensified between 4 and 12 h followed by a slower decreased in the last 12 h of fermentation. More specifically for FSp the reduction of phytate content during the first 12 h reached only 8% from the baseline, and during the following 12 h the reduction reached 51.8%. This reduction goes in line with the pH that remained above 6 during the first 12 h, a level that does not support optimal phytase activity. The pH dropped to 4.01 during the last 12 h explaining why most of the phytate reduction occurred during the later half of the 24-h fermentation. A similar pattern of phytate reduction was observed in a study where maize was spontaneously fermented for four days at 30°C, achieving a 61.5% reduction in phytates (29). Minor differences in rate of phytates reduction between our and theirs can be attributed to variations in temperature and the longer fermentation time. This slower reduction in the early stages of spontaneous fermentation can also be attributed to the slow decrease in the substrate’s pH, as discussed in the earlier section. Endogenous phytases of plant origin are most effective at degrading phytates in a pH range of 4.0–5.6. During fermentation the pH reduction and lactic acid production create the optimal environment for these phytases to become active and hydrolase the phytate molecules (20).

In our study, fermentations with starter cultures showed higher phytate reduction compared to spontaneous process, with FLp achieving a 65.3% reduction and FYLc reaching 68.7% in 24-h fermentation. For these two fermentations the fastest phytate reduction was detected between 4 and 12 h of fermentation, 47% out of the 65.3% for FLp and 57.5 out of the 68.7% for FYLc. Previous research has demonstrated that using specific starter cultures leads to greater phytate reduction than spontaneous fermentation. Hotz and Gibson (27) found a 49% phytate reduction when starter culture from a fermented beverage was added during maize fermentation at 25°C for 15 h, this was four times higher than the 12% reduction observed in spontaneous fermentation. Moreover, specific starter cultures such as *Lactobacillus amylovorus* and *Lentilactobacillus buchneri* have been shown to be effective phytase producers, achieving up to a 95.5% phytate reduction after 72 h of fermentation at 30°C (41).

Spontaneously fermented maize flour (FSp) showed the lowest phytate reduction, achieving only a 51.8% decrease from the baseline which was unfermented maize flour. This result may be due to the low specificity of microbial phytase from the endogenous microflora, as reported by Shimelis and Rakshit (32). In contrast, the highest phytate reduction occurred with the combined FLp-SG process, where fermentation with Lp299-inoculated pre-soaked and germinated maize led to an 85.6% reduction. The pre-treatments of soaking and germination enhanced phytate reduction by 20.3% (from 65.3% in FLp to 85.6% in FLp-SG). These findings highlight the effectiveness of pre-treatments in increasing phytate reduction before fermentation. Hotz and Gibson (27) also studied germination prior to fermentation, finding a 17% increase in phytate reduction when germination was used before natural fermentation of maize at 25°C for 48 h. Their work showed phytate reduction of 29% for flour from germinated maize grains, compared to 12% for unprocessed maize flour fermented for 96 h. However, their overall lower phytate reduction rates may have been influenced by the lower fermentation temperature (25°C). Our results align more closely with those reported by Khetarpaul and Chauhan (42) who reported an 88.3% reduction in phytate content by fermenting pre-germinated pearl millet flour (germinated at 30°C for 24 h) with *Saccharomyces cerevisiae* var. *diasticus*, *Saccharomyces cerevisiae*, *Levilactobacillus brevis*, and *Limosilactobacillus fermentum* at 30°C for 72 h. Among the factors which can accelerate or decelerate phytate reduction during fermentation of plant-based food, phytase activity is the most important factor that depends on the pH of substrate. Other factors include the microbial species, temperature and presence of anti-nutrients (43). Reducing phytate content, whether to a small or large extent, is beneficial for human nutrition as it decreases the risk of forming insoluble mineral complexes and improves mineral absorption in the gut.

Moreover, we found no significant difference in the effectiveness of FLp and FYLc fermentations for phytate reduction, indicating that yogurt containing viable lactic bacteria is as effective as the probiotic strain Lp299 as a starter culture. Overall, higher phytate reduction was achieved by incorporating pre-fermentation treatments such as soaking and germination before yogurt fermentation. This opens the possibility of applying this process at the household level. However, several challenges must be addressed before this can be fully realized, which we discuss extensively at the end of the discussion section.

### 4.4 Effect of soaking, germination fermentation on mineral content and estimated mineral bioavailability

The estimated bioavailability of minerals such as iron, zinc, and calcium are often calculated using the molar ratios of phytate to mineral because the presence of phytates significantly affects the absorption and utilization of these minerals in the human body. The molar ratio of phytate to minerals reflects the degree to which phytates can bind and prevent the absorption of essential minerals. The mechanism behind the molar ratios is funded by the fact that phytates are negatively charged molecules that strongly bind to positively charged minerals like iron, zinc, and calcium. These bonds form insoluble complexes, which prevent the minerals from being absorbed in the small intestine (44). The more phytate there is in relation to the mineral, the greater the likelihood of mineral-phytate complex formation, reducing the mineral’s availability for absorption. Thus, lower molar ratios generally indicate better potential for mineral absorption, making this calculation important for dietary planning and food processing aimed at improving the nutritional value of plant-based foods.

In this study, we found slight to non-significant changes in minerals contents of the fermented, soaked and germinated maize flours, results that agree with the findings of Castro-Alba et al. (18, 23) and Ayub et al. (16). In those studies, fermentation of pseudocereals did not have a significant effect modifying iron, zinc and calcium content of the raw and fermented pseudocereals. In our study a significant reduction of zinc was only found in FLp-SG and attributed to the soaking process used prior to this fermentation. This loss may be linked to the leaching effect of soluble mineral fractions in soaking water that was removed. Referring to the effect of lactic acid fermentation in improving minerals bioavailability of maize flours, we proposed Lp299 fermentation in continuum of maize kernel soaking and germination as the alternative that brings down the Phy: Zn ratio to a threshold level of 7.77 which is below 15 (desirable value). This result is similar with the results reported in a study in cassava fermentation, where the Phy:Zn ratio was improved from 16.31 to 1.71 after 48 h spontaneous fermentation at 25°C (45). Improvement of estimated bioavailability of zinc and iron in maize flour proves the efficiency of fermentation in phytate reduction and adds up to nutritional advantages.

Since application of plant—based fermented flours in food product development and food value addition is increasing, fermented maize flour can help improve essential minerals intake and absorption. Considering the context of developing countries where maize is a staple cereal, practicing soaking, germination and lactic acid fermentation in combination can alleviate the burden of micronutrient deficiencies and malnutrition.

### 4.5 Effect of fermentation on protein contents

Our results on protein contents of the fermented flours are nearly consistent with the results of Ogodo et al. (46) where 48 h fermentation of maize flour with lactic acid bacterial consortium improved protein contents from 9.44 to 12.9%. In another study, protein contents of maize flour were increased from 29.7 to 43.5% during various fermentations maize (47). Protein increases during fermentation have been attributed to several biochemical and microbiological processes that occur during fermentation. Microorganisms such as bacteria and yeast carbohydrates and other nutrients present in the substrate, they also consume nitrogen sources (aminoacids and peptides) to synthesize their own proteins, leading to an increase in bacterium biomass (48). Proteolytic activity has also been reported during fermentation, as this process involves the action of various enzymes, including proteases produced by the microorganism and the cereal itself. Proteolytic enzymes break down complex proteins into simpler peptides and free aminoacids, which may lead to an increase in protein content (49). Cell wall degradation led by the enzymes produced during fermentation has been reported (50). Some proteolytic enzymes can break down the aleurone layer’s cell wall structure, thus facilitating the release of proteins that are trapped within the bran of cereals. Thus, the synthesized proteolytic enzymes improve protein digestibility, and the changes in amino acid profile have positive effects increasing the overall nutritional quality of cereals. In this study, a slight increase in protein contents of during FYLc may be linked to the protein contents of the yoghurt used as inoculum for flour fermentation. Fermentation led improvement in protein content and quality of the maize flour is nutritionally advantageous and can further help to improve the palatability and texture of maize-based food products.

Along with protein, other nutrients in the substrate, such as starch and sugar, may also undergo significant changes during fermentation, warranting further research attention. It was reported that fermentation led to the breakdown of starch into simpler, more digestible sugars, and the reduction of sugars as microorganisms use them for energy. Besides proteolytic enzymes, amylolytic enzymes are also produced during fermentation. These amylases break down complex carbohydrates (starch) into simpler sugars (for example, maltose, and glucose), this process is called saccharification (51). Thus, fermented foods will usually result in lower glycemic index than their unfermented counterparts, which may have significant nutritional and health benefits. However, the specific changes depend on the type of microorganisms involved and the fermentation conditions.

We have noted in our previous studies of quinoa fermentation (23, 52) that fermentation can significantly affect the sensory characteristics, altering their taste, texture, aroma, and appearance. During fermentation, microbial activity breaks down complex carbohydrates and proteins, leading to the production of various compounds that influence sensory attributes such as taste. Overall, while fermentation enhances the nutritional value of cereals, it can also introduce complex sensory challenges that may either enhance or detract from consumer acceptability, depending on cultural preferences and intended use of the product. In our previous studies, we optimized the time of fermentation and concluded that the longest the fermentation time the more undesirable sensory characteristics are developed. For quinoa fermentation, we reduced the fermentation time from 48 to 9 h and added roasting after fermentation. This significantly improved the sensory characteristics of the fermented quinoa flour (23). We acknowledge that sensory is very important for consumer acceptability, while in the present study we reduced the fermentation time from 48 to 24 h, and we (4 people) in the research group evaluated basic characteristics such as aroma and taste, which seem to be acceptable. Further comprehensive studies should explore how different fermentation conditions - such as time, temperature, and microbial strains - affect these sensory properties. In the study conducted by Annan et al. (53) was highlighted the importance of the effect of different starter cultures in traditional African maize fermentation. It was shown that fermented maize dough in Ghana called “Koko” was preferred after spontaneous fermentation than after the use of a combination of starter cultures. Conversely, the sensory characteristics of the Nigerian “Ogi” prepared from fermented maize slurry were improved when starter cultures of *Levilactobacillus brevis* and *Saccharomyces cerevisiae* were used during fermentation (54). Understanding these changes will help optimize fermentation processes for improved consumer satisfaction and product development, especially in diverse cultural contexts where sensory preferences may vary.

By promoting local food processing techniques such as soaking, germination, and fermentation, families can improve the nutritional value of staple foods like maize without requiring expensive or complex technology. The extend by which this adoption could improve nutritional situation should be explored. Countries like Rwanda, where fermented foods are already part of their dietary habits and traditions are an important niche to start fermentation programs, where lactic bacteria found in commercial yogurt can be used as a starter culture. Fermentation is a traditional practice in many cultures, which makes its adoption more likely when compared to more modern, unfamiliar technologies. While modern food processing technologies such as ultrasound, pulsed electric fields, and enzymatic treatments offer promising alternatives for reducing phytates, fermentation remains a highly effective, affordable, and culturally adaptable method. It should not be dismissed as a “primitive” technology, as its potential to combat micronutrient deficiencies is considerable.

However, it is important to acknowledge the challenged associated with the widespread adoption of fermentation in household settings. Locations-specific fermentation conditions need to be validated, these regarding factors such as temperature, humidity and time, which can vary from one country to another. Further research and policy support and national programs are fundamental to develop detailed guidelines for home use of fermentation. The guidelines would offer households easy-to-follow instructions that ensure optimal nutrient enhancement while maintaining food safety. Widespread adoption of home fermentation can have profound social and economic impacts in developing countries, contributing to economic empowerment and improved public health. Furthermore, promoting home fermentation supports food sovereignty by empowering communities to rely on their traditional food systems rather than external technologies.

### 4.6 Limitations of the study

This study primarily focused on the nutritional effects of fermentation without considering the potential impact on sensory characteristics such as taste, texture, and aroma. Sensory properties such as aroma and taste were evaluated only by the research group. As these sensory attributes significantly influence consumer acceptability, the findings may not fully represent the practical implications of fermented products in real-world food systems. Future studies should incorporate sensory evaluation to provide a more comprehensive understanding of the acceptability of fermented foods. Another limitation of the study is the number of replicates used, two replicates of processing and two replicates of analysis, was relatively low, which may limit the statistical power and generalizability of the results. A larger sample size would enhance the reliability of the findings and allow for more robust conclusions. Further research on the field of fermentation should also include a throughout investigation on the impact of fermentation on the content and bioavailability of other important nutrients (starch, sugars) and bioactive compounds such as polyphenols.

## 5 Conclusion

This study demonstrates that simple processing techniques such as soaking, germination and fermentation effectively reduced phytates in maize flour. These methods have the potential to be applied at household level. For instance, FYLc, which uses yoghurt as starter culture, provided comparable results to the fermentation with Lp299. We also found that pre-treatments like soaking and germination used prior fermentation helped to further reduce the phytate content in maize. Reducing phytate reduction in this staple crop significantly improves the estimated bioavailability of essential minerals like iron and zinc which are crucial for addressing nutritional deficiencies in vulnerable populations. Additionally, the fermentation processes also increased the protein content in maize flour, which is particularly relevant in the current shift toward more protein-rich plant-based alternatives.

These findings underscored the potential of applying fermentation at the household level as a cost-effective dietary strategy to enhance protein content and improve mineral absorption, especially in populations vulnerable to protein and micronutrient deficiencies. The widespread adoption of household fermentation could improve nutrition security in developing regions. Moreover, well-document fermentation methods hold the potential to inform and shape national nutrition policies and programs aimed at combating malnutrition and micronutrient deficiencies.

## Data Availability

The raw data supporting the conclusions of this article will be made available by the authors, without undue reservation.
